# Supplementary material for: Prevalence of dental caries in the primary, mixed and permanent dentitions in Nigeria: A systematic review and meta-analysis
Source: PLoS One. 2026 Jun 1;21(6):e0349112. doi: 10.1371/journal.pone.0349112 (PMC13225390; doi:10.1371/journal.pone.0349112)
Supplement: S2 File — (PDF) [file pone.0349112.s002.pdf]

Appendix I

Search Strings

PubMed

((("dental caries"[MeSH Terms] OR "tooth decay"[MeSH Terms] OR "caries" OR "tooth caries" OR "tooth decay") AND ("primary teeth"[MeSH Terms] OR "deciduous teeth"[MeSH Terms] OR "milk teeth" OR "baby teeth" OR "permanent teeth"[MeSH Terms] OR "mixed dentition"[MeSH Terms] OR "mixed dentition")) AND ("Nigeria"[MeSH Terms] OR "Nigeria")) AND ("prevalence"[MeSH Terms] OR "burden of disease"[MeSH Terms] OR "severity of illness index"[MeSH Terms] OR "incidence"[MeSH Terms] OR "epidemiology"[MeSH Terms] OR "demography"[MeSH Terms])

CINAHL

((MH "Dental Caries" OR TX "Tooth Decay" OR TX "Caries" OR TX "Tooth Caries" OR TX "Tooth Decay") AND (MH "Primary Teeth" OR MH "Deciduous Teeth" OR TX "Milk Teeth" OR TX "Baby Teeth" OR MH "Permanent Teeth" OR MH "Mixed Dentition" OR TX "Mixed Dentition")) AND (TX "Nigeria") AND (MH "Prevalence" OR TX "Burden of Disease" OR MH "Severity of Illness Index" OR MH "Incidence" OR MH "Epidemiology" OR MH "Demography")

SCOPUS

(TITLE-ABS-KEY("dental caries") OR TITLE-ABS-KEY("tooth decay") OR TITLE-ABS-KEY("caries") OR TITLE-ABS-KEY("tooth caries") OR TITLE-ABS-KEY("tooth decay")) AND (TITLE-ABS-KEY("primary teeth") OR TITLE-ABS-KEY("deciduous teeth") OR TITLE-ABS-KEY("milk teeth") OR TITLE-ABS-KEY("baby teeth") OR TITLE-ABS-KEY("permanent teeth") OR TITLE-ABS-KEY("mixed dentition")) AND (TITLE-ABS-KEY("nigeria")) AND (TITLE-ABS-KEY("prevalence") OR TITLE-ABS-KEY("burden of disease") OR TITLE-ABS-KEY("severity of illness index") OR TITLE-ABS-KEY("incidence") OR TITLE-ABS-KEY("epidemiology") OR TITLE-ABS-KEY("demography"))

WoS

(TS=("dental caries" OR "tooth decay" OR "caries" OR "tooth caries" OR "tooth decay") AND TS=("primary teeth" OR "deciduous teeth" OR "milk teeth" OR "baby teeth" OR "permanent teeth" OR "mixed dentition") AND CU=("Nigeria" OR "Nigerian") AND TS=("prevalence" OR "burden" OR "severity" OR "incidence" OR "epidemiology" OR "population" OR "demographics"))

Search Strategy

PubMed Search Strategy

Search Terms:

Dental Caries and Tooth Decay:

"dental caries"[MeSH Terms]

"tooth decay"[MeSH Terms]

"caries"

"tooth caries"

"tooth decay"

Types of Teeth:

"primary teeth"[MeSH Terms]

"deciduous teeth"[MeSH Terms]

"milk teeth"

"baby teeth"

"permanent teeth"[MeSH Terms]

"mixed dentition"[MeSH Terms]

"mixed dentition"

Location:

"Nigeria"[MeSH Terms]

"Nigeria"

Epidemiological Concepts:

"prevalence"[MeSH Terms]

"burden of disease"[MeSH Terms]

"severity of illness index"[MeSH Terms]

"incidence"[MeSH Terms]

"epidemiology"[MeSH Terms]

"demography"[MeSH Terms]

Scopus Search Strategy

Search Terms:

Dental Caries and Tooth Decay:

"dental caries"

"tooth decay"

"caries"

"tooth caries"

"tooth decay"

Types of Teeth:

"primary teeth"

"deciduous teeth"

"milk teeth"

"baby teeth"

"permanent teeth"

"mixed dentition"

Location:

"nigeria"

Epidemiological Concepts:

"prevalence"

"burden of disease"

"severity of illness index"

"incidence"

"epidemiology"

"demography"

Web of Science Search Strategy

Search Terms:

Dental Caries and Tooth Decay:

“dental caries”

“tooth decay”

“caries”

“tooth caries”

“tooth decay”

Types of Teeth:

“primary teeth”

“deciduous teeth”

“milk teeth”

“baby teeth”

“permanent teeth”

“mixed dentition”

Location:

“Nigeria”

“Nigerian”

Epidemiological Concepts:

“prevalence”

“burden”

“severity”

“incidence”

“epidemiology”

“population”

“demographics”

CINAHL Search Strategy

Search Terms:

Dental Caries and Tooth Decay:

"Dental Caries" (Headings)

"Tooth Decay" (Keyword)

"Caries" (Keyword)

"Tooth Caries" (Keyword)

"Tooth Decay" (Keyword)

Types of Teeth:

"Primary Teeth" (Headings)

"Deciduous Teeth" (Headings)

"Milk Teeth" (Keyword)

"Baby Teeth" (Keyword)

"Permanent Teeth" (Headings)

"Mixed Dentition" (Headings)

"Mixed Dentition" (Keyword)

Location:

"Nigeria" (Geographic Location)

Epidemiological Concepts:

"Prevalence" (CINAHL)

"Burden of Disease" (Keyword)

"Severity of Illness Index" (CINAHL)

"Incidence" (CINAHL)

"Epidemiology" (CINAHL)

"Demography" (CINAHL)
