# Supplementary material for: Prevalence of dental caries in the primary, mixed and permanent dentitions in Nigeria: A systematic review and meta-analysis
Source: PLoS One. 2026 Jun 1;21(6):e0349112. doi: 10.1371/journal.pone.0349112 (PMC13225390; doi:10.1371/journal.pone.0349112)
Supplement: S1 Table — (PDF) [file pone.0349112.s004.pdf]

Supplementary File 4_JBI ASSESSMENT

S/No	First Author (Year)	Title	Study type	Risk of bias	Rating inconsistency	Rating indirectness	Rating imprecision	Assessing publication bias	Quality of evidence grades
1.	Adekoya-Sofowora, et al., 2006 [33]	Dental caries in 12-year-old suburban Nigerian school children	Cross-Sectional	Moderate	Moderate	Moderate	High	Moderate	Low
2.	Adeniyi, et al., 2009 [32]	Dental caries occurrence and associated oral hygiene practices among rural and urban Nigerian pre-school children	Cross-Sectional	Low	Moderate	Low	High	Moderate	Low
3.	Adeniyi, et al., 2012 [34]	Prevalence and Pattern of Dental Caries Among a Sample of Nigerian Public Primary School Children	Cross-Sectional	Low	Moderate	Moderate	High	Moderate	Moderate
4.	Adeniyi, et al., 2016 [31]	Dental Caries and Nutritional Status of School Children in Lagos, Nigeria - A Preliminary Survey	Cross-Sectional	High	Moderate	Moderate	High	Moderate	Low
5.	Adeniyi, et al., 2017 [35]	Self-Reported Dental Pain and Dental Caries Among 8–12-Year-Old School Children: An Exploratory Survey in Lagos, Nigeria	Cross-Sectional	Moderate	Moderate	Moderate	High	Moderate	Moderate
6.	Ajayi, et al., 2015 [43]	A 5-year retrospective study of rampant dental caries among adult patients in a Nigerian Teaching Hospital	Cohort	Low	Moderate	Low	High	High	Moderate
7.	Akhigbe, et al., 2022 [36]	Age-specific associations with dental caries in HIV-infected, exposed but uninfected and HIV-unexposed uninfected children in Nigeria	Cohort	Moderate	Moderate	Moderate	High	Moderate	Moderate
8.	Akinwonmi, et al., 2019 [37]	Oral health characteristics of children and teenagers with special health care needs in Ile-Ife, Nigeria.	Cross-Sectional	Moderate	Moderate	Moderate	High	Moderate	Moderate
9.	Akinyamoju, et al., 2018 [38]	Dental Caries and Oral Hygiene Status: Survey of Schoolchildren in Rural Communities, Southwest Nigeria	Cross-Sectional	Low	Moderate	Moderate	High	Moderate	High

10.	Aliyu, et al., 2019 [39]	Prevalence of dental caries in children with chronic heart disease	Cross-Sectional	Moderate	Moderate	Moderate	High	Moderate	Moderate
11.	Braimoh, et al., 2011 [40]	Caries and periodontal health status of prison inmates in Benin City, Nigeria.	Cross-Sectional	Low	Moderate	Low	High	Moderate	High
12.	Braimoh, et al., 2014 [41]	Caries Distribution, Prevalence, and Treatment Needs among 12–15-Year-Old Secondary School Students in Port Harcourt, Rivers State, Nigeria	Cross-Sectional	Moderate	Moderate	Low	High	Moderate	Low
13.	Chukwumah, et al., 2015 [42]	Impact of dental caries and its treatment on the quality of life of 12- to 15-year-old adolescents in Benin, Nigeria	Cross-Sectional	Low	Moderate	Moderate	High	Moderate	Low
14.	Dedeke, et al., 2014 [44]	Findings from a study in a defined urban population in South-western Nigeria using the PUFA index	Cross-Sectional	Low	Moderate	Low	High	Moderate	Low
15.	Denloye, et al., 2005 [45]	A Study of dental caries prevalence in 12–14-year-old school children in Ibadan, Nigeria	Cross-Sectional	Low	Moderate	Low	High	Moderate	High
16.	Denloye, et al., 2012 [46]	Oral health status of children seen at a pediatric neurology clinic in a tertiary hospital in Nigeria	Cross-Sectional	Moderate	Moderate	Low	High	Moderate	Very low
17.	Eigbobo, et al., 2017 [47]	Dental caries experience in primary school pupils in Port Harcourt, Nigeria	Cross-Sectional	Moderate	Moderate	Moderate	High	Moderate	High
18.	Ekowmenhenhen, et al., 2019 [48]	Adult Dental Caries Experience: A Rural-Urban Comparison in South-western Nigeria	Cross-Sectional	Moderate	Moderate	Low	High	Low	High
19.	El Tantawi, et al., 2021 [49]	Association between mental health, caries experience and gingival health of adolescents in sub-urban Nigeria	Cross-Sectional	Low	Moderate	Low	Low	Low	High
20.	Folayan, et al., 2012 [54]	Caries incidence in a cohort of primary school students in Lagos State, Nigeria followed up over a 3 years period	Cohort	Moderate	Moderate	Low	Low	Low	Very low
21.	Folayan, et al., 2015 [53]	Prevalence, and early childhood caries risk indicators in preschool children in suburban Nigeria	Cross-Sectional	Moderate	Moderate	Low	Low	Low	High
22.	Folayan, et al., 2020 [52]	Malnutrition, enamel defects, and early childhood caries in	Cross-Sectional	Low	Moderate	Low	Low	Low	High

		preschool children in a sub-urban Nigeria population							
23.	Folayan, et al., 2020 [51]	Validation of maternal report of early childhood caries status in Ile-Ife, Nigeria	Cross-Sectional	Low	Moderate	Moderate	Low	Low	High
24.	Folayan, et al., 2022 [50]	Risk indicators for dental caries, and gingivitis among 6–11-year-old children in Nigeria: a household-based survey	Cross-Sectional	Low	Moderate	Moderate	Low	Low	Moderate
25.	Iyun, et al., 2014 [55]	Prevalence and pattern of early childhood caries in Ibadan, Nigeria	Cross-Sectional	Moderate	Moderate	Moderate	Moderate	Low	Moderate
26.	Kolawole, et al., 2016 [57]	Digit Sucking Habit and Association with Dental Caries and Oral Hygiene Status of Children Aged 6 Months to 12 Years Resident in Semi-Urban Nigeria	Cross-Sectional	Low	Moderate	Moderate	Low	Low	High
27.	Kolawole, et al., 2019 [56]	Association between malocclusion, caries and oral hygiene in children 6 to 12 years old resident in suburban Nigeria	Cross-Sectional	Moderate	Moderate	Moderate	Low	Low	Moderate
28.	Lawal, et al., 2017 [58]	Dental caries experience and treatment needs of an adult female population in Nigeria.	Cross-Sectional	Moderate	Moderate	Moderate	Low	Moderate	Moderate
29.	Lawal, et al., 2019 [20]	Impact of Untreated Dental Caries on Daily Performances of Children from Low Social Class in an Urban African Population: The Importance of Pain	Cross-Sectional	Low	Moderate	Moderate	Low	Moderate	Moderate
30.	Nnawuihe, et al., 2016 [59]	An assessment of dental caries and periodontal disease burden in selected primary and secondary school children in Edo State, Southern – Nigeria	Cross-Sectional	Low	Moderate	Moderate	Low	Moderate	Moderate
31.	Nnawuihe, et al., 2021 [60]	Oral Disease Burden amongst Residents of an Internally Displaced Persons Camp in Nigeria	Cross-Sectional	Moderate	Moderate	Moderate	Low	Moderate	Moderate
32.	Ogbeide, et al., 2022 [61]	Prevalence of Dental Caries Among Children and Young Adults with Disabilities Attending a Special Needs School in Sokoto, Nigeria	Cross-Sectional	Moderate	Moderate	Moderate	Low	Moderate	Moderate

33.	Okoli, et al., 2021 [62]	Prevalence of common oral diseases among Senior Secondary School students in Enugu State, Nigeria	Cross-Sectional	Low	Moderate	Low	Moderate	Moderate	Moderate
34.	Okolo, et al., 2022 [63]	Dental Caries Prevalence, Severity, and Pattern Among Male Adolescents in Kano, Nigeria	Cross-Sectional	Low	Moderate	Low	Moderate	Moderate	Moderate
35.	Olabisi, et al., 2015 [64]	Prevalence of dental caries and oral hygiene status of a screened population in Port Harcourt, Rivers State, Nigeria	Cross-Sectional	Moderate	Moderate	Low	Moderate	High	High
36.	Olatosi, et al., 2015 [66]	The prevalence of early childhood caries and its associated risk factors among preschool children referred to a tertiary care institution	Cross-Sectional	Moderate	Moderate	Low	Moderate	High	Moderate
37.	Olatosi, et al., 2020 [68]	Disparities in Caries Experience and Socio-Behavioral Risk Indicators Among Private School Children in Lagos, Nigeria	Cross-Sectional	Moderate	Moderate	Low	Moderate	Moderate	Moderate
38.	Olatosi, et al., 2022 [65]	Dental Caries Severity and Nutritional Status of Nigerian Preschool Children	Cross-Sectional	Moderate	Moderate	Low	Moderate	Moderate	Moderate
39.	Olatosi, et al., 2022 [67]	Dental caries and oral health: an ignored health barrier to learning in Nigerian slums: a cross sectional survey	Cross-Sectional	Moderate	Moderate	Low	Moderate	Moderate	High
40.	Onyejaka, et al., 2016 [69]	Risk Factors of Early Childhood Caries among Children in Enugu, Nigeria	Cross-Sectional	Moderate	Moderate	Low	Moderate	Moderate	Moderate
41.	Onyejaka, et al., 2021 [71]	Prevalence and Associated Factors of Dental Caries among Primary School Children in South-East Nigeria	Cross-Sectional	Moderate	Moderate	Low	Moderate	Moderate	High
42.	Onyejaka, et al., 2021 [70]	Relationship Between Socio-Demographic Profile, Parity and Dental Caries Among a Group of Nursing Mothers in Southeast, Nigeria	Cross-Sectional	Moderate	Moderate	Low	Moderate	Moderate	High
43.	Osuh, et al., 2022 [72]	Prevalence and determinants of oral health conditions and treatment needs among slum and	Cross-Sectional	Low	Moderate	Low	Moderate	Moderate	High

		non-slum urban residents: Evidence from Nigeria							
44.	Oyedele, et al., 2018 [75]	Impact of oral hygiene and socio-demographic factors on dental caries in a suburban population in Nigeria	Cross-Sectional	Low	Moderate	Low	Moderate	Moderate	Moderate
45.	Oyedele, et al., 2020 [74]	Dental Caries Experience and MIH in Children Pattern and Severity NJBCS.	Cross-Sectional	Moderate	Moderate	Low	Moderate	Moderate	Moderate
46.	Oyeparo, et al., 2021 [73]	Association between dental caries, odontogenic infections, oral hygiene status and anthropometric measurements of children in Lagos, Nigeria	Cross-Sectional	Moderate	Moderate	Low	Moderate	Moderate	Moderate
47.	Ozeigbe, et al., 2013 [76]	Prevalence and clinical consequences of untreated dental caries using PUFA index in suburban Nigerian school children	Cross-Sectional	Low	Moderate	Moderate	Moderate	High	low
48.	Sofola, et al., 2014 [77]	Changes in the prevalence of dental caries in primary school children in Lagos State, Nigeria	Cohort	Moderate	Moderate	Moderate	High	High	low
49.	Soroye, et al., 2016 [78]	Oral health status, knowledge of dental caries aetiology, and dental clinic attendance: A comparison of secondary school students in the rural and urban areas of Lagos	Cross-Sectional	Moderate	Moderate	Moderate	High	High	low
50.	Sowole, et al., 2007 [79]	Dental caries pattern and predisposing oral hygiene related factors in Nigerian preschool children	Cross-Sectional	Moderate	Moderate	Moderate	High	High	Very low
51.	Umeizudike, et al., 2019 [80]	Oral health status and treatment needs of internally displaced persons	Cross-Sectional	Moderate	Moderate	Moderate	High	High	Moderate
52.	Uthman, et al., 2018 [81]	Prevalence of dental caries in public and private primary schools in llorin South Local Government Area of Kwara State, Nigeria	Cross-Sectional	Low	Moderate	Moderate	High	Moderate	Moderate
