# Supplementary material for: Prevalence of dental caries in the primary, mixed and permanent dentitions in Nigeria: A systematic review and meta-analysis
Source: PLoS One. 2026 Jun 1;21(6):e0349112. doi: 10.1371/journal.pone.0349112 (PMC13225390; doi:10.1371/journal.pone.0349112)
Supplement: S2 Table — (PDF) [file pone.0349112.s005.pdf]

Supplementary File 5: GRADE Rating Quality of Evidence

S/No	First Author (Year)	Q1 Was the sample frame appropriate to address the target population?	Q2 Were the study participants sampled in an appropriate way?	Q3 Was the sample size adequate?	Q4 Were the study subjects and the setting described in detail?	Q5 Was the data analysis conducted with sufficient coverage of the identified sample?	Q6 Were valid methods used for the identification of the condition?	Q7 Was the condition measured in a standard, reliable way for all participants?	Q8 Was there appropriate statistical analysis?	Q9 Was the response rate adequate, and if not, was the low response rate managed appropriately?	Score (%)	Quality Assessment
1.	Adekoya-Sofowora, et al., 2006 [33]	1	1	0	1	0	1	0	0	1	55.6	Moderate
2.	Adeniyi, et al., 2009 [32]	1	1	1	1	1	1	0	1	1	88.9	Low
3.	Adeniyi, et al., 2012 [34]	1	1	1	1	1	0	1	1	1	88.9	Low
4.	Adeniyi, et al., 2016 [31]	0	1	0	0	1	0	1	0	1	44.4	High
5.	Adeniyi, et al., 2017 [35]	1	1	1	0	0	1	0	0	1	55.6	Moderate
6.	Ajayi, et al., 2015 [43]	1	1	1	1	1	1	0	1	1	88.9	Low
7.	Akhigbe, et al., 2022 [36]	1	1	1	1	0	1	0	1	0	66.7	Moderate
8.	Akinwonmi, et al., 2019 [37]	1	1	1	1	0	0	1	0	1	66.7	Moderate
9.	Akinyamoju, et al., 2018 [38]	1	1	1	0	1	1	1	1	1	88.9	Low
10.	Aliyu, et al., 2019 [39]	1	0	0	0	1	1	1	1	1	66.7	Moderate

11.	Braimoh, et al., 2011 [40]	1	1	1	0	1	1	1	1	1	88.9	Low
12.	Braimoh, et al., 2014 [41]	1	1	1	1	0	0	1	1	1	77.8	Moderate
13.	Chukwumah, et al., 2015 [42]	1	1	1	1	1	1	0	1	1	88.9	Low
14.	Dedeke, et al., 2014 [44]	1	1	1	1	1	0	1	1	1	88.9	Low
15.	Denloye, et al., 2005 [45]	1	1	1	1	1	0	1	1	1	88.9	Low
16.	Denloye, et al., 2012 [46]	1	1	1	1	0	1	1	0	1	77.8	Moderate
17.	Eigbobo, et al., 2017 [47]	1	1	1	1	1	1	0	1	0	77.8	Moderate
18.	Ekowmenhenhen, et al., 2019 [48]	1	1	1	1	1	0	0	1	1	77.8	Moderate
19.	El Tantawi, et al., 2021 [49]	1	1	1	1	1	1	1	0	1	88.9	Low
20.	Folayan, et al., 2012 [54]	1	1	1	1	1	1	1	0	1	88.9	Moderate
21.	Folayan, et al., 2015 [53]	1	1	1	1	1	1	1	0	1	88.9	Moderate
22.	Folayan, et al., 2020 [52]	1	1	1	1	1	1	1	1	1	100.0	Low
23.	Folayan, et al., 2020 [51]	1	1	1	1	1	1	0	1	1	88.9	Low
24.	Folayan, et al., 2022 [50]	1	1	1	0	1	1	1	1	1	88.9	Low
25.	Iyun, et al., 2014 [55]	1	1	1	0	1	1	1	1	1	88.9	Moderate

26.	Kolawole, et al., 2016 [57]	1	1	1	1	1	0	1	1	1	88.9	Low
27.	Kolawole, et al., 2019 [56]	1	1	1	1	1	0	0	1	1	77.8	Moderate
28.	Lawal, et al., 2017 [58]	1	1	1	1	1	1	0	0	1	77.8	Moderate
29.	Lawal, et al., 2019 [20]	1	1	1	1	1	0	1	1	1	88.9	Low
30.	Nnawuihe, et al., 2016 [59]	1	1	1	1	1	1	0	1	1	88.9	Low
31.	Nnawuihe, et al., 2021 [60]	1	1	1	0	1	1	1	0	1	77.8	Moderate
32.	Ogbeide, et al., 2022 [61]	1	1	1	0	1	0	1	1	1	77.8	Moderate
33.	Okoli, et al., 2021 [62]	1	1	1	1	1	1	0	1	1	88.9	Low
34.	Okolo, et al., 2022 [63]	1	1	1	1	1	1	0	1	1	88.9	Low
35.	Olabisi, et al., 2015 [64]	1	1	1	1	1	0	1	0	1	77.8	Moderate
36.	Olatosi, et al., 2015 [66]	1	1	1	1	0	0	1	1	1	77.8	Moderate
37.	Olatosi, et al., 2020 [68]	1	1	1	1	1	0	0	1	1	77.8	Moderate
38.	Olatosi, et al., 2022 [65]	1	1	1	1	1	1	0	0	1	77.8	Moderate
39.	Olatosi, et al., 2022 [67]	1	1	1	1	1	1	0	0	1	77.8	Moderate
40.	Onyejaka, et al., 2016 [69]	1	1	1	1	1	0	1	0	1	77.8	Moderate

41.	Onyejaka, et al., 2021 [71]	1	1	1	1	1	0	1	0	1	77.8	Moderate
42.	Onyejaka, et al., 2021 [70]	1	1	1	1	0	1	1	0	1	77.8	Moderate
43.	Osuh, et al., 2022 [72]	1	1	1	1	1	1	0	1	1	88.9	Low
44.	Oyedele, et al., 2018 [75]	1	1	1	1	1	1	0	1	1	88.9	Low
45.	Oyedele, et al., 2020 [74]	1	1	1	1	1	0	0	1	1	77.8	Moderate
46.	Oyeparo, et al., 2021 [73]	1	1	1	1	1	0	0	1	1	77.8	Moderate
47.	Ozeigbe, et al., 2013 [76]	1	1	1	1	1	1	1	0	1	88.9	Low
48.	Sofola, et al., 2014 [77]	1	1	1	1	1	1	0	0	1	77.8	Moderate
49.	Soroye, et al., 2016 [78]	1	1	1	1	0	0	1	1	1	77.8	Moderate
50.	Sowole, et al., 2007 [79]	1	1	1	1	0	1	0	1	1	77.8	Moderate
51.	Umeizudike, et al., 2019 [80]	1	1	1	1	1	0	0	1	1	77.8	Moderate
52.	Uthman, et al., 2018 [81]	1	1	1	1	0	1	1	1	1	88.9	Low
