# Supplementary material for: Prevalence of dental caries in the primary, mixed and permanent dentitions in Nigeria: A systematic review and meta-analysis
Source: PLoS One. 2026 Jun 1;21(6):e0349112. doi: 10.1371/journal.pone.0349112 (PMC13225390; doi:10.1371/journal.pone.0349112)
Supplement: Attachment — Submitted filename: Response to the Editor_PLOS One.pdf [file pone.0349112.s008.pdf]

Response to the Editor

PONE-D-24-23202R1
Prevalence of Dental Caries in the Primary, Mixed, and Permanent Dentitions in Nigeria: A Systematic Review and Meta-analysis

Dear Editor,

Thank you for your feedback and for providing the opportunity to address the concerns raised regarding the S3 File.

In response:

1. A revised S3 File has been prepared and uploaded. It now includes a comprehensive table listing all 108 studies identified during the literature search. The table provides:
    - A numbered list of all studies, clearly distinguishing the 52 included studies and the 56 excluded studies.
    - For the excluded studies, detailed reasons for exclusion are specified to enhance transparency and reproducibility.
2. There were no included studies that are unpublished.

Please confirm if the revised S3 File meets your requirements or if further clarifications are needed.

Thank you for your guidance, and I look forward to your feedback.

Sincerely,
Dr. Folahanmi Tomiwa Akinsolu
On behalf of the Authors
