# Supplementary material for: Prevalence of dental caries in the primary, mixed and permanent dentitions in Nigeria: A systematic review and meta-analysis
Source: PLoS One. 2026 Jun 1;21(6):e0349112. doi: 10.1371/journal.pone.0349112 (PMC13225390; doi:10.1371/journal.pone.0349112)
Supplement: Attachment — Submitted filename: Response to Academic Editor_Akinsolu.pdf [file pone.0349112.s010.pdf]

Response to Academic Editor

Comment 1

Cohort studies are included in the analysis. The objective is to determine the prevalence of dental caries in the primary, mixed, and permanent dentitions.

Response:
Thank you for this important observation. We carefully re-examined the study designs of all studies included in the meta-analysis and confirmed that only one retrospective/cohort-type study was included. This study reported extractable prevalence-related data at a defined assessment point relevant to the review objective and was therefore retained in the analysis.

To ensure that its inclusion did not bias the pooled estimate, we conducted a leave-one-out sensitivity analysis. The results showed that exclusion of this study did not materially alter the overall pooled prevalence, indicating that it did not exert a disproportionate influence on the findings.

We have clarified this in the Methods, Results, and Limitations sections of the revised manuscript to improve transparency.

Comment 2

Recheck the extracted values of prevalence and sample size of all studies included in the meta-analysis.

Response:
Thank you for this important comment. We conducted a comprehensive re-evaluation of all extracted data, including prevalence values, sample sizes, dentition categories, and study setting classifications for each study included in the meta-analysis.
