# Supplementary material for: Prevalence of dental caries in the primary, mixed and permanent dentitions in Nigeria: A systematic review and meta-analysis
Source: PLoS One. 2026 Jun 1;21(6):e0349112. doi: 10.1371/journal.pone.0349112 (PMC13225390; doi:10.1371/journal.pone.0349112)
Supplement: Attachment — Submitted filename: Reviewer PONE.docx [file pone.0349112.s006.docx]

**CORRECTION OF THE MANUSCRIPT (PONE-D-24-23202)**

PLOS ONE

I am submitting my comments regarding the manuscript titled "Prevalence of Dental Caries in the Primary, Mixed and Permanent Dentitions in Nigeria: A Systematic Review and Meta-analysis." The study is a systematic review of prevalence aimed at identifying the prevalence of dental caries in Nigeria across deciduous, mixed, and permanent dentitions. I believe this study is significant. However, I list some concerns that should be considered by the authors.

In the introduction, there is a need for a better justification of the study's publication. What is its importance to the scientific community? How does it benefit the population of Nigeria? Have other countries conducted similar analyses?

In the methodology, please include the research question and the acronym used to outline the study. To assess bias risk, I suggest using the Joanna Briggs Institute's tool. Additionally, I recommend using the GRADE checklist to assess the certainty of evidence.

In the results, the authors identify extreme prevalence percentages. These data should be interpreted cautiously considering the certainty of evidence from the included studies.

In the discussion, I suggest including the strengths and limitations of the study towards the end of this section. Additionally, all discussed points need to be referenced.

Decision: **major review**
