# Supplementary material for: Prevalence of dental caries in the primary, mixed and permanent dentitions in Nigeria: A systematic review and meta-analysis
Source: PLoS One. 2026 Jun 1;21(6):e0349112. doi: 10.1371/journal.pone.0349112 (PMC13225390; doi:10.1371/journal.pone.0349112)
Supplement: Attachment — Submitted filename: Response to Reviewers_Akinsolu.docx [file pone.0349112.s009.docx]

**Response to Reviewers**

We sincerely thank the Academic Editor and the Reviewers for their thorough evaluation of our manuscript and for the constructive comments provided. We have carefully revised the manuscript in response to all comments raised. Below, we provide a detailed point-by-point response.

**Additional Editor Comments**

1. The % symbol is repeated in each row in Table 3 under the column ‘Prevalence of dental caries’.

*Response: Thank you for this observation. We have revised Table 3 by removing the repeated “%” symbol in the 'Prevalence of dental caries' column to improve clarity and formatting consistency.*

2. Please provide high-resolution figures. Most of the figures are blurred and not clearly visible.

*Response: Thank you for this important comment. All figures have now been regenerated and uploaded in high resolution to ensure clarity and readability. In addition, following the reviewers’ suggestions, we reanalyzed the data using R (RStudio) for the meta-regression, Egger’s test, and sensitivity analyses. The updated, high-quality figures reflecting these analyses have been included in the revised manuscript.*

3. Comment on the publication bias considering the funnel plot. Provide the results of Egger’s test or Begg and Mazumdar’s test.

*Response: Thank you for this important suggestion. We performed Egger’s linear regression test to formally assess funnel plot asymmetry. The test did not indicate statistically significant evidence of small-study effects (t = –0.60, df = 33, p = 0.552). Although visual inspection of the funnel plot suggested some asymmetry, the substantial heterogeneity observed across studies (I² ≈ 97–99%) likely explains the dispersion rather than publication bias. The manuscript has been updated accordingly.*

4. Studies included in the meta-analysis from 2001 to 2023. The prevalence of dental caries varies over the years. How may this affect the pooled prevalence? Provide meta-regression findings.

*Response: Thank you for this important observation. To assess whether temporal variation influenced the pooled prevalence, we conducted a meta-regression analysis using publication year as a continuous moderator. The analysis demonstrated a positive but non-statistically significant association (coefficient = 0.0384, p = 0.139), indicating that publication year did not significantly explain heterogeneity in prevalence estimates. Therefore, pooling studies across the study period is unlikely to have substantially biased the overall prevalence estimate. The manuscript has been revised to include these findings.*

5. How was the sensitivity analysis conducted and what were the findings?

*Response: Thank you for this important suggestion. Sensitivity analyses were conducted using two approaches. First, a leave-one-out analysis was performed, whereby each study was sequentially excluded to assess its influence on the pooled estimate. The pooled prevalence remained stable across iterations, indicating that no single study unduly influenced the results. Second, a trim-and-fill procedure was applied to assess the impact of potential small-study effects. The adjusted pooled prevalence remained comparable to the original estimate (0.22; 95% CI: 0.17–0.26), suggesting that the overall findings are robust.*

**Reviewer #3 Comments**

1. The English is weak, sentences are long, and formatting is inconsistent.

*Response: We appreciate this observation. The entire manuscript has been thoroughly revised to improve grammar, clarity, and readability. Long and complex sentences have been rewritten, and formatting inconsistencies, including font style and size discrepancies, have been corrected to ensure uniform presentation throughout the manuscript.*

2. The search dates are not consistent between the abstract and methods section.

*Response: Thank you for identifying this inconsistency. The discrepancy resulted from earlier draft versions. The search dates have now been standardized throughout the manuscript. The final search period is consistently reported as January 2001 to December 2023 in both the abstract and the methods section.*

3. The discussion should mention important quantitative results.

*Response: Thank you for this constructive suggestion. The Discussion section has been substantially revised to explicitly highlight the key quantitative findings, including the overall pooled prevalence (17%; 95% CI: 14%–21%), subgroup estimates by dentition type and geographical setting, and the extent of heterogeneity (I² values).*

4. Please double-check the reference list formatting.

*Response: The reference list has been carefully reviewed and reformatted to ensure full consistency with the journal’s citation style. In-text citations and references have been cross-checked for completeness and accuracy.*

**Reviewer #4 Comments**

1. Assess the evolution of prevalence over time using chronological ordering or meta-regression.

*Response: We thank the reviewer for this valuable suggestion. To formally assess temporal trends, we conducted a meta-regression analysis using publication year as a continuous moderator variable. The analysis demonstrated a positive but non-statistically significant association (coefficient = 0.0384, p = 0.139), indicating that publication year did not significantly explain between-study heterogeneity.*

2. Funnel plot asymmetry suggests possible publication bias. Egger’s test should clarify.

*Response: We agree that visual inspection of the funnel plot suggested some asymmetry. To formally assess small-study effects, we conducted Egger’s linear regression test, which did not demonstrate statistically significant evidence of funnel plot asymmetry (t = –0.60, df = 33, p = 0.552). Given the substantial heterogeneity (I² ≈ 97–99%), the asymmetry likely reflects genuine variability rather than publication bias. The manuscript has been revised accordingly.*

3. Differences between dentition modes and settings have overlapping confidence intervals.

*Response: We agree that the confidence intervals overlap across dentition types and settings. A formal test for subgroup differences was conducted and did not demonstrate statistically significant differences (χ² = 1.53, df = 2, p = 0.47 for dentition type). The Discussion has been revised to clarify that these represent descriptive patterns rather than statistically confirmed differences.*

4. Consider a three-level meta-analysis due to clustering by authors.

*Response: We thank the reviewer for this thoughtful suggestion. Although two authors contributed multiple studies, each study was conducted in independent populations without overlapping samples. Therefore, the effect sizes represent independent observations. Given the absence of dependency at the dataset level, we believe a three-level meta-analysis model is not required. This clarification has been added to the limitations section.*

We hope that the revisions made have adequately addressed all concerns raised by the Editor and Reviewers. We sincerely appreciate the time and expertise invested in reviewing our work.
